# Supplementary material for: Evidence supported by Mendelian randomization: impact on inflammatory factors in knee osteoarthritis
Source: Front Med (Lausanne). 2024 May 28;11:1382836. doi: 10.3389/fmed.2024.1382836 (PMC11165061; doi:10.3389/fmed.2024.1382836)
Supplement: Supplementary file 1 [file Data_Sheet_1.ZIP › data/GCST90274808_5e6/808.leaveoneout.pdf]

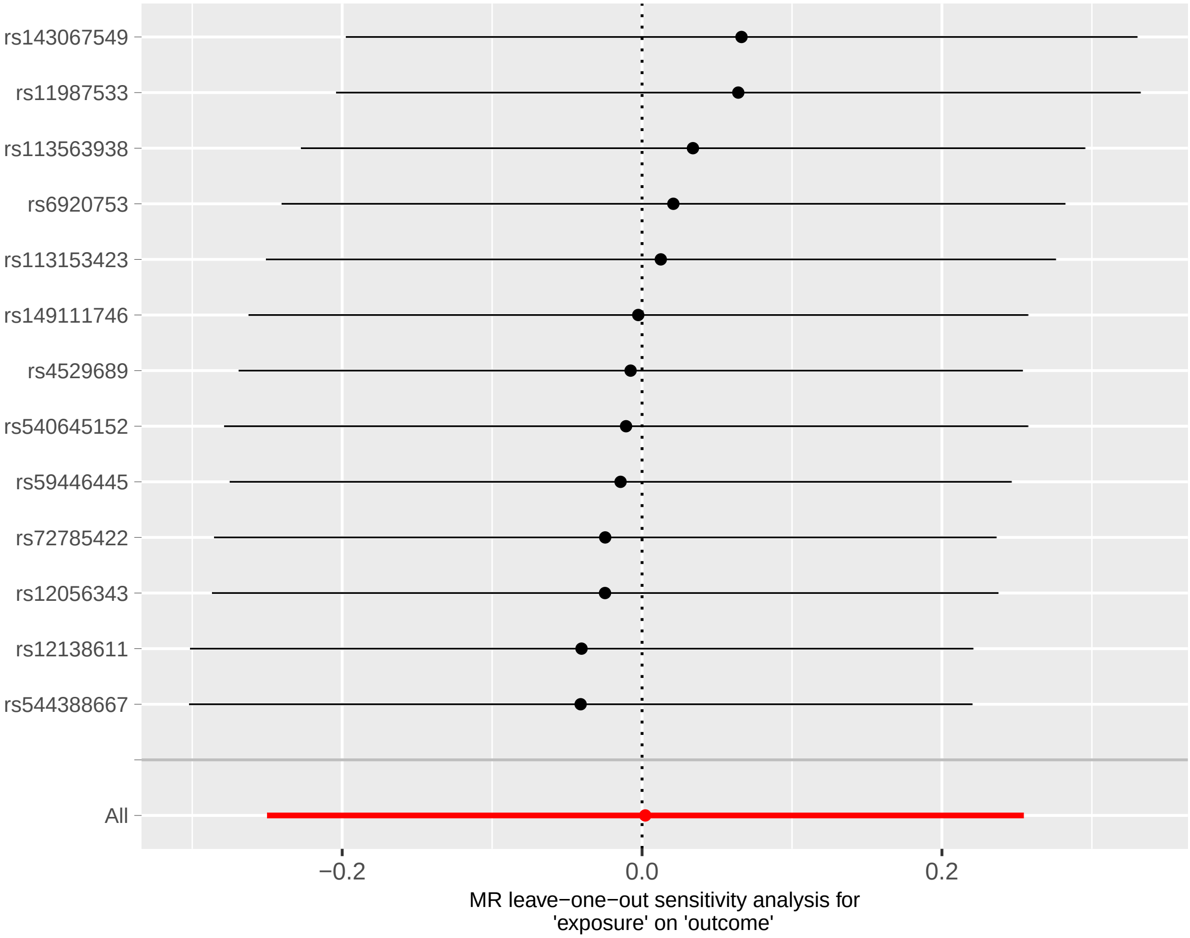

rs143067549
rs11987533
rs113563938
rs6920753
rs113153423
rs149111746
rs4529689
rs540645152
rs59446445
rs72785422
rs12056343
rs12138611
rs544388667
All
−0.2
0.0
0.2
MR leave−one−out sensitivity analysis for
'exposure' on 'outcome'
